# Ultra‐Wide‐Field Noninvasive Imaging Through Scattering Media Via Physics‐Guided Deep Learning

**DOI:** 10.1002/advs.75390

**Published:** 2026-05-07

**Authors:** Lintao Peng, Mingwei He, Jeff Zhu, Sujit K. Sahoo, Liheng Bian, Cuong Dang

**Affiliations:** ^1^ School of Electrical and Electronic Engineering Nanyang Technological University Singapore Singapore; ^2^ State Key Laboratory of Environment Characteristics and Effects for Near‐space & State Key Laboratory of CNS/ATM Beijing Institute of Technology Beijing China; ^3^ School of Electrical Sciences Indian Institute of Technology Goa Goa India

**Keywords:** memory effect, noninvasive Imaging, physics‐guided deep learning, scattering media, untra wide field of view

## Abstract

Noninvasive imaging through scattering media is crucial for diverse applications but remains constrained by a narrow field of view (FOV). Although recent learning‐based methods have a larger FOV, they often require large‐scale real experimental datasets and struggle when the FOV is far beyond the optical memory effect (OME). Here, we propose a physics‐guided adaptive dual‐domain diffusion model for ultra‐wide‐field noninvasive imaging through scattering media, namely UNI‐Net. Specifically, we first develop a physical scattering imaging model to synthesize large‐scale pre‐training data, thereby reducing dependence on real experimental datasets. Second, to maximize the utilization of speckle information, we partition each speckle pattern into multi‐channel patches to guide the diffusion process. Third, we propose a spatial‐channel parallel attention block to model the spatial sparsity and inter‐channel similarity of speckle patches with linear complexity. Extensive experiments show that our method cuts reliance on real experimental data by an order of magnitude and achieves a PSNR of 31.23 dB at a 41× OME range in complex scenes, which is 49.5% higher than existing approaches while requiring significantly lower computational and memory costs. Even at an extreme 164× OME range where other methods fail, it still reliably reconstructs complex scenes with a PSNR of 27.21 dB.

## Graphical abstract

We propose a physics‐guided adaptive dual‐domain learning method for ultra‐wide‐field noninvasive imaging through scattering media, namely UNI‐Net. Our method not only reduces the requirement for real experimental data by an order of magnitude but also enables clear imaging of complex scenes with an ultra‐large field of view, which is 164 times the OME range.

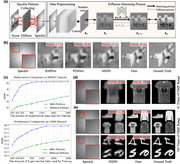

## 1 Introduction

Imaging within or through strongly scattering media is critical across various fields, including biomedical [1, 2, 3], physics [4], and environmental monitoring [5]. In such media, light undergoes scattering events, resulting in complex speckle patterns that obscure the original object information. By analyzing speckle or other diffraction features, recent developments can recover obscured objects. Early studies [6, 7, 8] attempted reconstruction by extracting ballistic photons. However, strongly scattering media significantly attenuate these photons, leading to diminished signal strength and degraded image quality [9]. Wavefront shaping [10, 11, 12, 13] and transmission‐matrix measurement [14, 15] can enhance image quality but require elaborate optical setups and often invasive measurements, rendering them unsuitable for dynamic scattering environments. Diffuse optical tomography [16, 17] and time‐of‐flight imaging [6, 16, 18] offer alternative strategies, yet their resolution remains several orders of magnitude below the optical diffraction limit. Speckle‐correlation imaging [19, 20], which leverages the optical memory effect (OME), requires only one speckle pattern and uses phase retrieval algorithms [21, 22] to reconstruct the concealed object. Nonetheless, its field of view is inherently limited by the OME range. Moreover, limitations in algorithms and camera performance, together with noise and sample complexity, often lead to reconstruction failures or convergence to artifact‐laden solutions.

In recent years, researchers have explored many data‐driven methods to learn the mapping between speckle patterns and hidden objects. Horisaki et al. [23] proposed an SVR‐based algorithm trained on a large‐scale speckle dataset for single‐shot imaging through scattering media. However, machine‐learning approaches often rely on hand‐crafted priors and extensive parameter tuning, which result in poor generalization, suboptimal reconstruction quality, and slow inference speed. More recently, convolutional neural networks (CNNs) [24] have been applied to this inverse problem, yielding significant progress. Existing CNN‐based scattering imaging methods can be divided into two categories. (1) end‐to‐end CNN‐based methods [25, 26] which directly map speckle patterns to images; (2) physical‐prior enhanced methods [27, 28] that embed physical priors into the CNN network. Shuai Li et al. [25] pioneered the use of CNNs for scattering imaging with “IDiffNet,” adopting a negative Pearson correlation coefficient (NPCC) loss for training. Subsequently, Yunzhe Li et al. [26] introduced a statistical “one‐to‐all” CNN that generalizes across diverse diffusers within the same class and delivers high‐fidelity object reconstructions. Nevertheless, these exclusively data‐driven methods neglect physical priors, leading to overfitting and limited generalizability. To address this limitation, Shuo Zhu et al. [28] introduced speckle‐correlation priors for speckle preprocessing, extracting statistical invariants across conditions and enabling the network to learn more discriminative features. Fu Liu et al. [27] extended this approach by leveraging speckle–scene autocorrelation as a physical prior and proposing a self‐supervised training scheme for network adaptation to diverse scattering conditions. However, autocorrelation potentially throws much information away and may fail to leverage all the information contained in speckles. Moreover, because the physical constraints are applied only during preprocessing, network training remains predominantly data‐driven, potentially leading to inefficient utilization of physics‐based priors.

Recently, Transformer‐based imaging approaches [29, 30] have shown significant promise in seeing through scattering media. By leveraging the self‐attention mechanism [31], Transformers can capture long‐range dependencies and non‐local similarities, thereby mitigating key limitations of CNNs. However, these methods still face several main challenges. First, in the global attention Transformer [31], the computational complexity is quadratic to the spatial size, this burden is non‐trivial and sometimes unaffordable. Second, existing Transformer‐based methods ignore the physical prior that high‐frequency regions are harder to reconstruct than smooth regions, treating all areas equally and yielding suboptimal imaging results. Third, current learning‐based scattering imaging frameworks [25, 26, 27, 28, 32, 33, 34] predominantly utilize U‐Net architectures, framing reconstruction as an end‐to‐end image enhancement task that directly maps speckle inputs to high‐fidelity target images. Although these models perform well under mild to moderate degradation, their restoration capabilities under severe degradation remain fundamentally limited. Moreover, existing learning‐based scattering imaging methods [25, 26, 27, 28, 32, 33, 34] often require large volumes of real experimental data for training, which severely limits their practical deployment.

**FIGURE 1:**
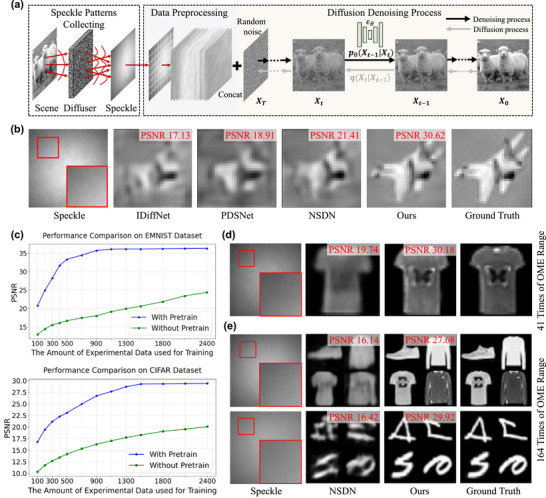
(a) The overview of our method. We first segment the captured speckle pattern into 64‐channel speckle patches that align with the spatial scale of the target scene. These patches are then used to guide the iterative denoising process in our proposed UNI‐Net. (b) Visualized experiment results of different techniques for imaging through scattering media with complex natural scenes. The compared methods include IDiffNet [25], PDSNet [32], NSDN [34], and our proposed method. (c) Extensive experimental results demonstrate that pretraining with simulated data generated by our proposed physical scattering imaging model can significantly improve reconstruction performance and reduce the reliance on real speckle data by an order of magnitude. For example, in the imaging experiment of the EMNIST dataset, using 200 pairs of real speckle‐target scene data pairs under the premise of pre‐training can achieve comparable reconstruction performance to training with 2400 pairs of real data without pretraining. (d) Our method enables high‐quality imaging of the target scene at 41 × the OME limit. (e) Even at 164× the OME limit, where conventional techniques fail to yield reconstructions, our method still delivers faithful reconstruction on the complex scenes.

To address these challenges, as shown in Figure 1a, we introduce the denoising diffusion probabilistic model [35] into imaging through scattering media, and propose a physics‐guided adaptive dual‐domain learning method for ultra‐wide‐field noninvasive imaging through scattering media, namely UNI‐Net. Our method not only reduces the requirement for real experimental data by an order of magnitude but also enables clear imaging of complex scenes with an ultra‐large field of view (FOV), which is 164 times the OME range. Specifically, we first develop a physical scattering imaging model to generate large‐scale synthetic training data, thereby reducing reliance on real experimental data. Second, to maximize information utilization in speckle patterns, we partition each acquired speckle pattern into multi‐channel patches at the spatial scale of the target scene (Figure 1a). Third, leveraging both the spatial‐sparsity prior of natural scenes and the inter‐channel similarity of multi‐channel speckle patches, we propose the spatial‐wise State Space Module (SSM) and channel‐wise SSM based on the Mamba model [36], and combine these two modules in parallel to form a spatial‐channel parallel attention block (SC‐block). Mamba is a recently proposed state‐space model that can be regarded as a linear computational complexity self‐attention module with a global receptive field and content‐based reasoning ability [37]. Leveraging this, the spatial‐wise SSM efficiently captures the global spatial receptive field with linear computational complexity, guiding the network to focus on information‐dense regions while avoiding unnecessary computation in sparse areas. Meanwhile, the channel‐wise SSM models inter‐channel similarities with linear complexity and capture long‐range dependencies across channels. The parallel design enables spatial and channel features to interact, promoting complementary feature fusion across channels. In addition, considering that the spatial regions with high‐frequency details are more difficult to reconstruct, we introduce a frequency‐wise loss (FWL) to narrow the frequency domain discrepancy. Dynamic FWL supervision forces the model to reconstruct fine‐grained frequencies and improves the imaging quality in regions with rich detail.

The superior performance of our UNI‐Net was demonstrated and compared with other techniques using EMNIST [38], Fashion‐MNIST [39] and CIFAR [40] datasets. As shown in Figure 1b, our method markedly outperforms existing approaches in reconstructing fine textures and structural details through unknown scattering media. Furthermore, Figure 1c shows that pretraining with simulated data generated from our physical scattering imaging model greatly improves the imaging performance and reduces reliance on real experimental measurements by an order of magnitude. In addition, our method facilitates ultra‐wide‐field imaging that extends orders of magnitude beyond the OME limit. With a FOV of 41× OME range, our UNI‐Net achieves an imaging PSNR of 31.23 dB on the Fashion‐MNIST, yielding a 49.5% improvement over prevailing methods while requiring lower computational and memory costs (Figure 1d). Crucially, even at a 164× OME range FOV, where conventional techniques fail to yield reconstructions, our UNI‐Net still delivers faithful reconstruction on the complex scenes with an imaging PSNR of 27.21 dB (Figure 1e).

## 2 Results

### 2.1 Experimental Setup and OME Range Measurement

**FIGURE 2:**
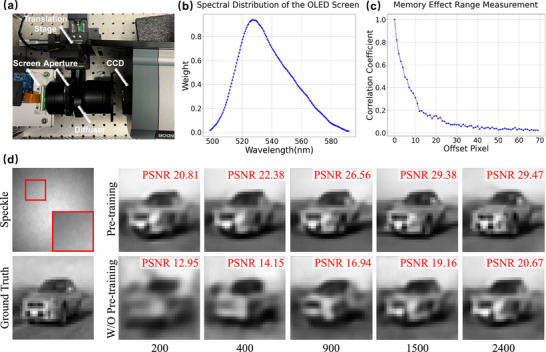
(a) The proof‐of‐concept scattering imaging system. (b) Spectral distribution characteristics of the OLED screen measured with a spectrometer. (c) The OME range of the proof‐of‐concept scattering imaging system. (d) Visualization of reconstruction results with and without (W/O) pre‐training under varying amounts of real data. The top row shows reconstruction results of the proposed model pre‐trained on simulated data, then fine‐tuned using different amounts of real data. The bottom row shows the results of the model trained from scratch with the same amount of real data. These results demonstrate that pre‐training significantly improves reconstruction quality and reduces the need for real experimental data by an order of magnitude.

To acquire real speckle patterns, we built a proof‐of‐concept setup for noninvasive imaging through scattering media. As illustrated in Figure 2a, the target scene is rendered on an OLED screen, and the emitted light first passes through a pinhole to define the aperture of imaging system and suppress stray illumination. Subsequently, the beam traverses a large‐area diffuser mounted on a motorized stage that undergoes 2D randomized translations. The nature randomness of the ground glass diffuser guarantees different scattering characteristics at different scattering regions, generating a unique point spread function (PSF) for each acquisition [41] and thereby enriching the dataset. Finally, the scattered light is captured by a CCD detector, and the recorded speckle patterns are fed into our model to facilitate high‐quality image reconstruction.

To measure the OME range of the optical system, we placed a point source as the center pixel of a 768×1024 pixel OLED display and recorded the corresponding speckle pattern. The target was subsequently translated one pixel to the right for each successive acquisition, and speckle patterns were captured, the correlation coefficient between the acquired speckles and the reference speckles (at the central position) was computed as Figure 2c. The correlation drops quickly after the point source shift of about 10 pixels then the dropping rate is slower. The correlation drops to half when the point source shifts 5 pixels on one side, which implies that our system's OME range is about 10 pixels. An object of 10×10 pixels will be completely within the OME region. An object of 32×32 pixels will be beyond the OME region, but still maintain the correlation of greater than 0.1.

### 2.2 Proposed Physical Imaging Model and Simulation Data Generation

The scattering process can be modeled as the convolution of the object O(x,y) with the system's shift‐invariant PSF,

(1) I(x,y)=O(x,y)∗PSF(x,y),

where I(x,y) is the recorded intensity on the imaging plane. For speckle simulation, the key is to construct a PSF that reflects the characteristics of the scattering medium, namely high‐frequency attenuation and random phase perturbation. Specifically, in the 2D frequency domain $\left(f_{x},f_{y}\right)$, we first determine the physical cutoff frequency $f_{c}=\frac{2\pi \mathrm{NA}}{\lambda }$ based on the central wavelength λ of the light and the numerical aperture (NA), and then simulate the system's band‐limited aperture using a circular binary function on a discrete grid,

(2) $$A\left(f_{x},f_{y}\right)=\left\{\begin{array}{cc} 1, & \sqrt{f_{x}^{2}+f_{x}^{2}}\leq f_{c}\\ 0, & \text{otherwise.} \end{array}\right.$$

To accurately locate the cutoff radius on a finite‐size FFT grid, we introduce the sampling frequency $F_{0}=\frac{\pi M}{P}$, here, M is the magnification of the optical system, and P is the physical pixel size of the camera. Thus, the frequency corresponding to the discrete grid point $\left(k_{x},k_{y}\right)$ is given by,

(3) $$f_{x},f_{y}=\left(k_{x}\frac{F_{0}}{N},k_{y}\frac{F_{0}}{N}\right),k_{x},k_{y}\in \left[-N,N\right].$$

Accordingly, we define a dimensionless scaling factor $\alpha =\frac{f_{c}}{F_{0}}$, such that the cutoff radius falls exactly at $\sqrt{k_{x}^{2}+k_{y}^{2}}=N\alpha$ on the grid. Here, N denotes half the grid size and determines the frequency domain resolution.

Next, phase perturbations $\phi (f_{x},f_{y})$ are applied to each frequency component within the band‐limited region to simulate phase randomization caused by multiple scattering in the medium, thereby yielding the complex frequency‐domain transfer function,

(4) $$\tilde{H}\left(f_{x},f_{y}\right)=A\left(f_{x},f_{y}\right)\cdot e^{j\phi (f_{x},f_{y})},$$

then the normalized spatial‐domain PSF can be obtained by applying the inverse fast Fourier transform (IFFT) and taking the squared magnitude,

(5) $$\mathrm{PSF}\left(x,y\right)=\frac{∥F^{-1}\left\{\tilde{H}\right\}\left(x,y\right){∥}^{2}}{\sum_{x,y}{∥F^{-1}\left\{\tilde{H}\right\}∥}^{2}}.$$

This process effectively transforms band‐limited high‐frequency information (cutoff frequency) and random phase components in the frequency domain into spatial‐domain speckle patterns, whose statistical properties closely mimic those produced by real scattering media. To accelerate computation, the calculation is performed in the frequency domain using the Fast Fourier Transform (FFT).

Furthermore, to more accurately approximate realistic imaging conditions, a synthetic light source comprising multiple spectral bands is employed in the simulation. Each spectral band $\lambda_{i}$ has its own cutoff frequency and phase perturbation characteristics due to wavelength differences, corresponding to distinct amplitude functions $A_{\lambda_{i}}\left(f_{x},f_{y}\right)$ and random phase distributions $\phi_{\lambda_{i}}\left(f_{x},f_{y}\right)$. Therefore, each spectral band has a PSF and corresponding speckle $I_{\lambda_{i}}\left(x,y\right)$. The final image resulting from the multi‐band synthetic source is obtained by a weighted summation of all band images, where the weights are given by the spectral distribution $S(\lambda_{i})$ (i.e., the relative intensity of each band), expressed as,

(6) $$I\left(x,y\right)=\sum_{i}^{N_{\lambda }}S\left(\lambda_{i}\right)I_{\lambda_{i}}\left(x,y\right).$$

This superposition process reflects the actual imaging scenario where multiple spectral bands simultaneously contribute to image formation in an optical system. To obtain the weight of each spectral band in the broadband light source, we measured the spectral distribution of the OLED screen using a spectrometer. The measurement results are shown in Figure 2b, where the weight of each spectral band corresponds to the weight $S(\lambda_{i})$ assigned to the speckle pattern generated by that band during simulation. Other key physical parameters of the physical imaging model were obtained by direct measurement on the proof‐of‐concept optical setup we built. Specifically, in our optical setup, the NA is 0.04 (see supplementary document for the method of measuring NA), the magnification factor is 1.4 and the physical size of each CCD pixel is 13.5 μm, so that each CCD pixel corresponds to a 9.64 μm patch on the object plane.

### 2.3 The Necessity of Pretraining With Simulated Data

Based on the proposed physical scattering imaging model, we generated large‐scale simulated datasets using the publicly available EMNIST [38] and CIFAR [40] benchmarks, which were subsequently employed to pre‐train our UNI‐Net. Specifically, we first measured the key physical parameters (including NA, optical magnification factor, and the weight of each spectral band in the light source) of our optical setup and then incorporated these parameters into our physical imaging model to enable realistic data simulation. To ensure data diversity, the PSF was updated every 50 image pairs during simulation. For each dataset, we generated 10 000 pairs of speckle‐ground truth images and split them into training and testing sets in a 9:1 ratio for pre‐training. Subsequently, we collected 2800 pairs of real speckle‐ground truth images for each dataset and divided them into training and testing sets (9:1) to fine‐tune and evaluate the UNI‐Net. The scattering media were moved to a new region (new scattering characteristics) every 50 images. Two different diffusers were used for training and testing sets to make sure that the testing dataset utilized unseen scattering media. These 2800 images are entirely distinct from the previously generated 10 000 simulated images.

To evaluate the effectiveness of the proposed physical scattering imaging model and the pre‐training strategy, we conducted comparative experiments under two experimental settings: with pre‐training and without pre‐training. Under the pre‐training setting, our UNI‐Net was first pre‐trained on a simulated dataset and then fine‐tuned using different amounts of real speckle‐ground truth image pairs. In contrast, under the setting without pre‐training, the UNI‐Net was trained directly on an equivalent amount of real‐world data from scratch. The experimental results are summarized in Figure 1c and Figure 2d. In addition, we report the detailed imaging performance (in terms of PSNR (dB) and SSIM) with and without pre‐training under different amounts of real data in the Supporting Information.

As shown in Figure 1c, pre‐training on simulated data significantly improves UNI‐Net's reconstruction performance and reduces its dependence on real data. Specifically, as shown in Figure 2d, the pre‐trained model achieved an imaging PSNR of 20.81 dB using only 200 real image pairs, whereas the model trained from scratch required 2 400 image pairs to achieve comparable performance. This demonstrates that pre‐training on simulated data generated by our physical scattering imaging model effectively reduces the requirement for real data by an order of magnitude. The visual results in Figure 2d also illustrate that pre‐training enables clearer and more accurate reconstructions even when trained with limited real experimental data. These results demonstrate the efficacy of the proposed physical scattering imaging model and confirm that pre‐training on simulated data followed by fine‐tuning on real data significantly enhances the model's generalizability in real‐world scenarios.

### 2.4 Imaging Performance Comparison

Most studies on imaging through scattering media are validated only on simple datasets such as MNIST, EMNIST [38], or facial images [42], which cover limited categories and simple structures, restricting the evaluation of generalizability to complex scenes. To assess our UNI‐Net's capability in reconstructing more complex and diverse scenes, we adopt the Fashion‐MNIST [39] and CIFAR [40] benchmarks. Fashion‐MNIST comprises various clothing items, whereas CIFAR contains natural images with richer textures and more intricate object structures. For all speckle acquisitions, the displayed scene was fixed at a size of 32×32 pixels. The compared methods are SVR [23], IDiffNet [25], PDSNet [32], DeepSCI [33], NSDN [34], and our proposed UNI‐Net. Among them, SVR is a machine‐learning‐based method. IDiffNet, PDSNet and NSDN are end‐to‐end CNN‐based methods that use a U‐Net to map speckle patterns to images directly. Deep‐SCI is a physical‐prior enhanced methods that embed speckle‐correlation priors into the CNN network.

All compared methods were implemented using the authors' official code or faithfully reimplemented according to the description in the paper. To ensure a fair comparison, prior to the comparative experiments, we train and test each reproduced network following the original paper's experimental settings to ensure it achieves the reported imaging performance. When conducting comparative experiments, all the methods involved in the comparison were trained and tested in the same hardware and software environment. We followed the parameter settings of the original paper of the comparative method and trained its network model until the loss function converged. Please refer to the Supporting Information for detailed reproduction and training procedures.

**FIGURE 3:**
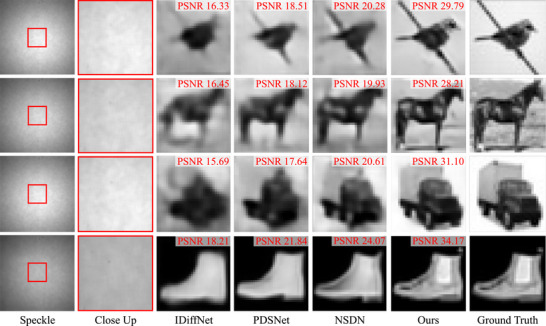
Visualized results for performance comparison of different techniques for imaging through unknown scattering media with complex scenes. The experiment is described in Table 1.

The experimental results are presented in Table 1 and Figure 3. Our UNI‐Net achieves the highest PSNR and SSIM, significantly outperforms all competing approaches, while requiring cheaper computational and memory costs. Visualizations in Figure 3 show that existing methods struggle to reconstruct complex scenes. These methods recover only coarse global structures, fail to reconstruct detailed textures and sharp edges, and often introduce artifacts. In contrast, our UNI‐Net accurately restores scene structure and faithfully reconstructs texture‐rich regions and sharp edges. This superior performance arises from three key innovations. First, we partition the full speckle pattern into multichannel speckle patches and use them as guidance within the UNI‐Net's iterative denoising process, thereby maximizing speckle‐information utilization. Second, the proposed SC‐block provides a global spatial receptive field and models long‐range inter‐channel dependencies with linear computational complexity, enabling the network to recover fine‐grained structures in complex images. Third, the FWL loss function reinforces the network's attention to challenging texture‐rich and edge regions, further enhancing image quality.

**TABLE 1:** Quantitative performance comparison of imaging through scattering media with complex scenes on the CIFAR and Fashion‐MNIST datasets. The compared methods include SVR [23], IDiffNet [25], PDSNet [32], DeepSCI [33], NSDN [34], and our proposed method. For fair comparison, all methods were first pre‐trained on the same simulated dataset, then fine‐tuned on the same real dataset, and finally evaluated on the same test set. Our method significantly outperforms existing approaches in both PSNR and SSIM metrics while requiring cheaper computational and memory costs.

Methods	CIFAR		Fashion MNIST		FLOPs(G)	#Param.(M)
	PSNR	SSIM	PSNR	SSIM		
SVR	12.37	0.402	16.38	0.461	—	—
IDiffNet	16.29	0.506	19.74	0.613	17.04	24.16
PDSNet	19.34	0.601	22.98	0.687	24.03	58.4
DeepSCI	18.16	0.564	22.17	0.645	35.62	46.34
NSDN	20.86	0.648	24.19	0.718	21.65	38.79
Ours	**29.41**	**0.895**	**33.79**	**0.922**	12.93	8.25

### 2.5 Imaging FOV Beyond Hundred Times of OME Range

Although the OME enables imaging through strongly scattering media, its inherently limited FOV significantly restricts its applicability in medical imaging and environmental sensing. Existing imaging methods [23, 25, 32, 33, 34] remain inadequate for producing accurate reconstructions when the object size substantially exceeds the OME range. This limitation arises because, as object dimensions far surpass the OME range, the PSF for each region becomes decorrelated to each other, i.e. we do not have a shift‐invariant PSF anymore, challenging all the traditional imaging methods. In addition, the speckle patterns become overly smoothed due to super‐position of uncorrelated speckles, making it difficult for U‐Net‐based end‐to‐end networks to extract sufficient features to reconstruct the accurate structural information. In contrast, our UNI‐Net leverages multi‐channel speckle patches as guiding information and iteratively reconstructs the target scene through multiple denoising steps, thereby maximizing the utilization of speckle information. We presented multiple target images of 64×64 and 128×128 pixels on the OLED screen, equivalent to approximately 41× and 164× of the OME range, respectively. Finally, we compared the imaging performance of IDiffNet [25], PDSNet [32], NSDN [34], and our UNI‐Net under both experimental conditions.

**FIGURE 4:**
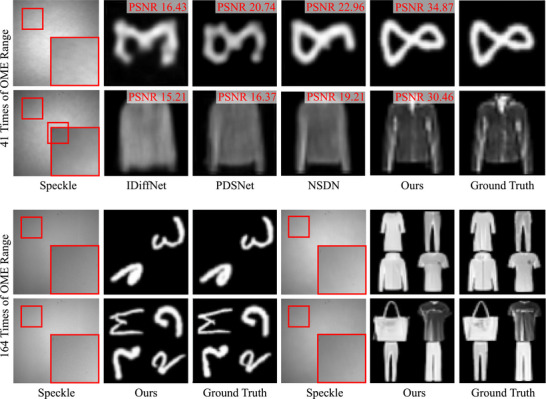
Visualized imaging performance comparison results of different techniques through unknown scattering media with FOVs extend 41× and 164× beyond the OME range. The experiment is described in Table 2.

Experimental results (Table 2 and Figure 4) clearly highlight the limitations of existing approaches and the robustness of our method in extreme scattering scenarios. Under the 41× OME range, IDiffNet fails to recover coherent shapes, producing blob‐like artifacts, while PDSNet and NSDN capture only rough contours at the cost of overly smoothed textures and fuzzy edges. By contrast, our method accurately reconstructs the overall structure of the target and clearly restores the local details on Fashion MNIST, which achieves 31.23 dB PSNR (a 49.5% boost over NSDN's 20.89 dB) and 0.879 SSIM (48.4% higher than NSDN's 0.592). A similar advantage is observed on EMNIST, where we reach 34.27 dB PSNR (+38.1%) and 0.926 SSIM (+34%). When pushed to the challenging 164× OME range, all baselines collapse (best PSNR ≤ 17.36 dB, SSIM ≤ 0.538), yielding severely distorted, unrecognizable outputs. In stark contrast, our method remains stable and faithful, successfully recovering legible digits on EMNIST (29.87 dB PSNR, 0.841 SSIM) and garments on Fashion‐MNIST (27.21 dB PSNR, 0.806 SSIM).

**TABLE 2:** Quantitative results of different methods under 41× and 164× OME ranges on EMNIST and Fashion‐MNIST datasets. For fair comparison, all methods were first pretrained on the same simulated dataset, then fine‐tuned on the same real dataset, and finally evaluated on the same test set. Our method significantly outperforms existing approaches in both PSNR and SSIM metrics, especially at 164× OME where other methods fail to reconstruct meaningful images.

Methods	41× OME range				164× OME range			
	EMNIST		Fashion MNIST		EMNIST		Fashion MNIST	
	PSNR	SSIM	PSNR	SSIM	PSNR	SSIM	PSNR	SSIM
SVR	15.97	0.476	14.26	0.456	13.69	0.437	12.87	0.429
IDiffNet	19.39	0.554	17.91	0.545	15.37	0.469	15.19	0.451
PDSNet	23.67	0.667	20.13	0.587	16.82	0.508	15.93	0.486
DeepSCI	21.32	0.596	18.73	0.551	16.31	0.496	15.42	0.472
NSDN	24.81	0.691	20.89	0.592	17.36	0.538	16.25	0.506
Ours	**34.27**	**0.926**	**31.23**	**0.879**	**29.87**	**0.841**	**27.21**	**0.806**

This improvement arises from our two‐stage approach of pre‐training on simulated data followed by fine‐tuning on real measurements, which significantly enhances the model's robustness under ultra‐wide‐field conditions. It is worth noting that our simulation data set uses shift‐invariant PSF (i.e. infinite OME range), the experimental data refines the model to incorporate the limited OME range. It also demonstrates that incorporating multi‐channel speckle intensity as physical supervision during each iteration of the diffusion model's denoising process effectively improves reconstruction fidelity. Moreover, segmenting the acquired speckle pattern into multi‐channel blocks substantially increases information utilization. Overall, the integration of simulation‐based pre‐training with multi‐channel speckle‐guided iterative denoising is central to the superior performance of our method.

### 2.6 Ablation Study

To demonstrate the effectiveness of each component, we conducted a series of ablation studies on the EMNIST dataset and the Fashion MNIST dataset. We consider several factors including the spatial‐wise SSM (SSSM) and channel‐wise SSM (CSSM) module in the SC‐block, the multi‐channel speckle block (MCSB) input strategy and the FWL function. The baseline (BL) model is derived by replacing our SC‐block with traditional convolutional block, and remove the MCSB strategy and the FWL function from our method. Serial and Parallel represent combining the CSSM and SSSM modules using serial and parallel design, respectively. The statistical experimental results are shown in Table 3.

**TABLE 3:** Break‐down ablation study. Models are trained and tested on the EMNIST and Fashion MNIST dataset, respectively.

BL	MCSB	SSSM	CSSM	Serial	Parallel	FWL	EMNIST		Fashion MNIST	
							PSNR	SSIM	PSNR	SSIM
✓							27.88	0.753	24.93	0.702
✓	✓						30.14	0.814	26.58	0.749
✓	✓	✓					31.75	0.857	28.12	0.792
✓	✓		✓				30.69	0.829	27.82	0.784
✓	✓	✓	✓	✓			32.89	0.871	29.29	0.825
✓	✓	✓	✓		✓		34.48	0.918	32.06	0.903
✓	✓	✓	✓		✓	✓	**36.25**	**0.949**	**33.79**	**0.922**

#### 2.6.1 Effectiveness of SC‐block

From Table 3, we observe that the removal of any component from the SC‐block leads to a degradation in performance, which demonstrates the effectiveness of each component in the SC‐block and the effectiveness of their combination. Moreover, we can see that using a parallel design to combine CSSM and SSSM results in better imaging performance than using a serial design. This is because in a serial design, the network processes spatial and channel features in successive steps, which makes these two types of features interact less. Conversely, the parallel design facilitates the interaction of spatial and channel features while maintaining linear complexity, thereby enabling the SC‐block to simultaneously capture spatial‐wise and channel‐wise global receptive fields, model the spatial sparsity and inter‐channel similarity of the multi‐channel speckle block input, and perform adaptive dual‐domain scattering imaging.

#### 2.6.2 Effectiveness of FWL

We calculate the log frequency distance (LFD) as a frequency‐level metric to evaluate the spectrum difference. The LFD has a logarithmic relationship with the frequency distance d(u,v) in Equation (7),

(7) $$\begin{array}{c} F_{LFD}=log\left[\frac{1}{HW}\left(\sum_{u=0}^{H-1}\sum_{v=0}^{W-1}\left|d\left(u,v\right)\right|\right)+1\right]. \end{array}$$

**FIGURE 5:**
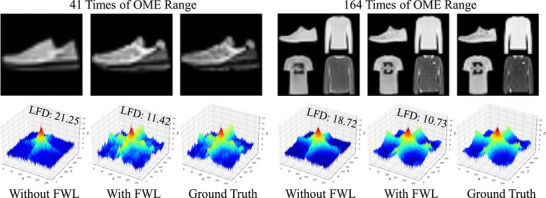
Frequency spectrum visualization with or without FWL under different OME ranges. The metric LFD is used to measure the frequency similarity. The smaller the LFD, the closer the reconstructed image is to the ground truth.

As shown in Figure 5, we visualize the 3D‐spectra reconstructed with or without the FWL and provide the corresponding LFD. It can be seen that the 3D‐spectra optimized with our proposed FWL exhibit more accurate frequency reconstruction and lower LFD, aligning the frequency statistics more closely with the ground truth. On the contrary, the frequency 3D‐spectra of the reconstructed image without FWL supervision lose a lot of frequency information, resulting in amplitude and phase distortions that manifest as blurred details and artifacts in the predicted image. Additionally, as shown in Table 3, the inclusion of FWL leads to an increase in the PSNR and SSIM scores of our method. This is because fine‐grained spectrum supervision can preserve more high‐frequency information that is difficult to synthesize, thereby achieving higher imaging performance. Moreover, since FWL does not change the network structure, the improvements achieved by FWL will not bring any additional memory and computational costs during testing.

#### 2.6.3 Effectiveness of the MCSB Strategy

To further evaluate the contribution of the MCSB strategy, we conducted ablation experiments by comparing models trained with and without MCSB. As shown in Table 3, removing the MCSB strategy results in a notable drop in PSNR and SSIM, demonstrating its significance in enhancing image reconstruction quality. This result stems from partitioning the acquired speckle pattern according to the optical magnification into multiple channels that match the target scale, thereby maximizing the utilization of speckle information and enabling the network to extract sufficient features for accurate scene reconstruction.

## 3 Conclusion

In this work, we address the dual bottlenecks of limited imaging FOV and heavy reliance on real speckle data in the existing learning‐based methods for noninvasive imaging through scattering media. To this end, we propose a physics‐guided adaptive dual‐domain diffusion model for ultra‐wide‐field noninvasive imaging through scattering media, namely UNI‐Net. Specifically, we first construct a physical scattering imaging model to generate synthetic data for pretraining, significantly reducing dependence on real experimental data. Second, we segment the speckle pattern into multi‐channel blocks at the same spatial scale as the target scene to guide the diffusion denoising process, thereby maximizing speckle‐information utilization. Third, we design a parallel spatial‐channel attention block based on the state‐space model, which captures global spatial attention and long‐range inter‐channel dependencies with linear complexity, thus enabling dual‐domain adaptive imaging. Finally, to guide the network to better focus on hard‐to‐reconstruct high‐frequency details, we introduce a frequency‐wise loss function. Experimental results demonstrate that pretraining with our physical scattering model reduces the requirement for real speckle data by an order of magnitude, which is crucial for the broader applications of noninvasive imaging through scattering media. Moreover, compared with existing scattering imaging methods, our approach achieves significantly improved imaging quality and can clearly reconstruct large targets beyond 164× the OME range.

Thanks to the above advantages, our proposed UNI‐Net has broad application potential in multiple fields. For example, in biomedical and clinical settings, the reduced reliance on large experimental datasets and the ability to recover fine structure through strongly scattering tissue make our method promising for noninvasive imaging (e.g., shallow subsurface visualization, intraoperative tissue assessment, and ex vivo/ in vitro studies where rapid model tuning is required). For live‐tissue and functional imaging, where sample motion and variable scattering frequently degrade conventional reconstructions, the combination of multichannel speckle guidance and frequency‐aware loss improves stability and detail recovery, facilitating higher‐throughput and more reproducible experiments. Beyond biomedicine, our UNI‐Net is well‐suited for environmental and remote sensing tasks in scattering atmospheres or turbid water, for industrial non‐destructive testing where hidden defects lie behind diffusive layers, and for non‐contact examination of cultural heritage objects where non‐destructive, wide‐field inspection is essential. In addition, our cross‐domain feature modeling and effective incorporation of physical priors provide new insights for advancing scattering imaging techniques.

It is important to note that when dividing the captured speckle pattern into multi‐channel speckle patches, the size and number of patches are not chosen arbitrarily. The speckle size is determined by measuring the magnification factor of the optical setup, and then calculating the pixel coverage of the target scene on the sensor. In our optical setup, the magnification is approximately 1; thus, for a target scene of size 64×64, the speckle pattern is segmented into patches of the same size (64×64 pixels). To determine the optimal number of patches, we experimented with 4, 8, 16, 64, and 128 patches. Reducing the number from 64 to 16 led to a 17% drop in reconstruction performance, while increasing it to 128 yielded less than a 1% improvement at the cost of quadrupling the model size. To balance imaging quality and model complexity, we ultimately selected 64 speckle patches.

In this work, the maximum imaging resolution we achieve is 128×128, corresponding to approximately 164× the OME range. When the resolution is further increased to 256×256 (656× the OME range), we encounter two challenges that prevent high‐fidelity imaging at that scale. First, owing to the CCD's 2048×2048 resolution, a 256×256 target permits at most 16 non‐overlapping 256×256 speckle patches rather than the 64 channels available at lower resolutions. As a result, the imaging network cannot extract sufficient scene features from the multichannel speckle inputs, which limits the final reconstruction quality. Second, as shown in Figure 4, the speckle pattern for a 128×128 scene ( 164× OME range) is significantly smoother than that for a 64×64 scene (41× OME range). When the scene size increases to 256×256, the speckle becomes excessively smooth, making it difficult for the network to extract sufficient discriminative features for accurate reconstruction. The first issue can probably be addressed by increasing the CCD resolution or altering the magnification of the optical system. The second issue may be mitigated by enhancing the contrast of speckle patterns or by designing networks with stronger feature extraction capabilities. In future work, we will focus on addressing these challenges to further expand the FOV of noninvasive imaging through scattering media while maintaining reconstruction fidelity.

## 4 Method

### 4.1 The Framework of Our Imaging Model

**FIGURE 6:**
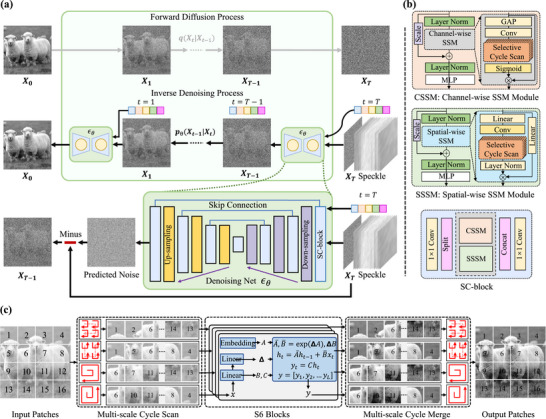
(a) Detailed structure of the proposed UNI‐Net. (b) We combine the Spatial‐wise SSM and Channel‐wise SSM in parallel to form an SC‐block. The Spatial‐wise SSM is built based on our proposed multi‐scale SSM, which can capture the global spatial receptive field with linear complexity, guiding the network to focus on information‐dense regions while avoiding unnecessary computation in sparse areas. Meanwhile, the Channel‐wise SSM models inter‐channel similarities with linear complexity and capture long‐range dependencies across channels. The parallel design enables spatial and channel features to interact, promoting complementary feature fusion across channels, thereby enabling dual‐domain adaptive imaging. (c) The framework of our proposed multi‐scale SSM. Given the input data X, the multi‐scale SSM first unfolds input patches into sequences along multiple distinct traversal paths (i.e., multi‐scale cycle scan), processes each patch sequence using a separate S6 block in parallel, and subsequently reshapes and merges the resultant sequences to form the output map (i.e., feature‐merge).

Most learning‐based scattering‐imaging methods use U‐Net [43] to map speckle patterns to clean images in an end‐to‐end manner. While effective under mild or moderate degradation, they struggle with severe scattering due to limited information extraction. To address this, we incorporate a denoising diffusion probabilistic model (DDPM) [35] into scattering imaging. As shown in Figure 6a, our UNI‐Net progressively transforms Gaussian noise into the target image distribution through DDPM‐based refinement steps guided by the speckle input, using a U‐shaped denoising backbone that removes noise across multiple scales. Given paired training data $D={\left\{x_{i},y_{i}\right\}}_{i=1}^{N}$ drawn from an unknown conditional distribution p(y∣x), our goal is to approximate this distribution via a stochastic reverse process that maps a speckle image x to its ground truth y. Conditional DDPM achieves this by starting from Gaussian noise $y_{T}∼N\left(0,1\right)$ and iteratively generating $\left(y_{T-1},\text{…},y_{0}\right)$ through learned conditional transitions $p_{\theta }\left(y_{\left(t-1\right)}|y_{t},x\right)$, eventually yielding $y_{0}∼p\left(y|x\right)$. These transitions are trained to invert a forward diffusion process $q(y_{t}|y_{t-1})$ that gradually adds Gaussian noise. The denoising model $f_{\theta }$ learns to estimate and remove this noise from intermediate samples, enabling effective conditional image reconstruction during inference. We first give an overview of the forward diffusion process, and then discuss how our denoising model $f_{\theta }$ is trained and used for inference.

### 4.2 Forward Diffusion Process

The forward diffusion process is a Markovian chain, which gradually adds Gaussian noise to a target image $y_{0}$ over T iterations:

(8) $$\begin{array}{cc} q(y_{1:T}|y_{0}) & =\prod_{t=1}^{T}q\left(y_{t}|y_{t-1}\right),\\ q(y_{t}|y_{t-1}) & =N(y_{t}|\sqrt{\alpha_{t}}y_{t-1},\left(1-\alpha_{t}\right)I), \end{array}$$

where the scalar parameters $\alpha_{1:T}$ ($0<\alpha_{t}<1$) are hyperparameters, which determine the variance of the noise added at each iteration. The $y_{t-1}$ is attenuated by $\sqrt{\alpha_{t}}$ to ensure that the variance of the random variables remains bounded as t→∞.

Importantly, one can characterize the distribution of $y_{t}$ given $y_{0}$ by marginalizing out the intermediate steps as,

(9) $$\begin{array}{c} q\left(y_{t}|y_{0}\right)=N\left(y_{t}|\sqrt{\gamma_{t}}y_{0},\left(1-\gamma_{t}\right)I\right) \end{array}$$

where $\gamma_{t}=\prod_{i=1}^{t}\alpha_{i}$. Furthermore, with some algebraic manipulation and completing the square, one can derive the posterior distribution of $y_{t-1}$ given $\left(y_{0},y_{t}\right)$ as,

(10) $$\begin{array}{cc} & q\left(y_{t-1}|y_{0},y_{t}\right)=N\left(y_{t-1}|\mu ,\sigma^{2}I\right)\\ & \mu =\frac{\sqrt{\gamma_{t-1}}\left(1-\alpha_{t}\right)}{1-\gamma_{t}}y_{0}+\frac{\sqrt{\alpha_{t}}\left(1-\gamma_{t-1}\right)}{1-\gamma_{t}}y_{t}\\ & \sigma^{2}=\frac{\left(1-\gamma_{t-1}\right)\left(1-\alpha_{t}\right)}{1-\gamma_{t}} \end{array}$$

This posterior is useful for parameterizing the reverse chain and constructing the variational lower bound. Next, we detail how to train a neural network to reverse the Gaussian diffusion process.

### 4.3 Training the Denoising Model

The noisy image $\tilde{y}$ can be regarded as the sum of the clean target image $y_{0}$ and the Gaussian noise ε to be removed,

(11) $$\tilde{y}=\sqrt{\gamma }y_{0}+\sqrt{1-\gamma }\varepsilon ,\;\varepsilon ∼N\left(0,I\right),$$

This definition of the noisy image $\tilde{y}$ aligns with the marginal distribution of noisy images at different steps of the forward diffusion process in Equation (9).

Previous conditional diffusion models use class labels or text as guidance. We instead segment the captured speckle pattern into multi‐channel speckle blocks x to guide the reverse process, and input them with the noisy image $\tilde{y}$ into the denoising network $f_{\theta }$. In addition to those two inputs, the denoising model $f_{\theta }\left(x,\tilde{y},\gamma \right)$ also takes a sufficient statistic of the noise variance γ as input and is trained to predict the noise vector ε. By conditioning on the scalar γ, we enable the model to be aware of the noise level, similar to [44]. The proposed training objective for $f_{\theta }$ is:

(12) $$E_{\left(x,y\right)}E_{\varepsilon ,\gamma }{∥f_{\theta }\left(x,\underset{\tilde{y}}{\underset{︸}{\sqrt{\gamma }y_{0}+\sqrt{1-\gamma }\varepsilon }},\gamma \right)-\varepsilon ∥}_{p}^{p}$$

where ε∼N(0,I),(x,y) is sampled from the training dataset, p∈{1,2}, and γ∼p(γ). The distribution of γ has a big impact on the quality of the model and the generated outputs.

### 4.4 Inverse Denoising Process

During inference, the goal is to generate the target image $y_{0}$ conditioned on the multi‐channel speckle input x. This is achieved through a learned reverse diffusion process, which iteratively transforms a sample from a simple prior distribution into a clean image sample. Specifically, the process begins with a sample $y_{T}∼N\left(0,1\right)$, representing pure Gaussian noise, and proceeds through a series of denoising steps to recover $y_{0}$. The reverse process is formulated as a reverse Markov chain parameterized by a neural network $f_{\theta }$, which predicts the noise component present in the current noisy image at each time step. The full generative model is defined as,

(13) $$p_{\theta }\left(y_{0:T}|x\right)=p\left(y_{T}\right)\prod_{t=1}^{T}p_{\theta }\left(y_{t-1}|y_{t},x\right),$$

where the prior is $p\left(y_{T}\right)=N\left(y_{T}|0,I\right)$, and each transition is modeled as a Gaussian distribution,

(14) $$p_{\theta }\left(y_{t-1}|y_{t},x\right)=N\left(y_{t-1}|\mu_{\theta }\left(x,y_{t},\gamma_{t}\right),\sigma_{t}^{2}I\right).$$

To approximate the denoized image $y_{0}$, the network $f_{\theta }$ predicts the noise ε added at time step t. Using this predicted noise, we compute the estimated clean image,

(15) $$\left.{\hat{y}}_{0}=\frac{1}{\sqrt{\gamma_{t}}}\left(y_{t}-\sqrt{1-\gamma_{t}}\right.f_{\theta }\left(x,y_{t},\gamma_{t}\right)\right).$$

This estimate is then used to parameterize the mean of the reverse transition distribution, following the derivation of the posterior in the forward diffusion process,

(16) $$\mu_{\theta }\left(x,y_{t},\gamma_{t}\right)=\frac{1}{\sqrt{\alpha_{t}}}\left(y_{t}-\frac{1-\alpha_{t}}{\sqrt{1-\gamma_{t}}}f_{\theta }\left(x,y_{t},\gamma_{t}\right)\right),$$

and the variance of $p_{\theta }\left(y_{t-1}|y_{t},x\right)$ is set as $\left(1-\alpha_{t}\right)$. Following this parameterization, each iteration of iterative refinement under our model takes the form,

(17) $$y_{t-1}\leftarrow \frac{1}{\sqrt{\alpha_{t}}}\left(y_{t}-\frac{1-\alpha_{t}}{\sqrt{1-\gamma_{t}}}f_{\theta }\left(x,y_{t},\gamma_{t}\right)\right)+\sqrt{1-\alpha_{t}}\varepsilon_{t},$$

where $\varepsilon_{t}∼N\left(0,I\right)$. This iterative refinement procedure allows the model to gradually remove noise and recover the data distribution, leveraging the conditional information from the input x. Compared to autoregressive models, this method enables parallel computation across pixels and maintains a constant number of steps regardless of image resolution.

### 4.5 Detailed Network Structure

To simultaneously capture the spatial sparsity of the target scene and the inter‐channel similarity of multi‐channel speckle blocks with linear computational complexity, we propose a parallel spatial‐channel attention block (SC‐block) grounded in the state‐space model [45]. Built upon this SC‐block, we construct a U‐shape network as the denoising network in the reverse diffusion process.

Moreover, existing diffusion models [35, 46] typically require 1‐2k inference steps, rendering them inefficient for high‐resolution generation tasks. To improve efficiency, our UNI‐Net conditions directly on the noise variance γ rather than the diffusion step t, allowing flexible control over the number of inference steps and the noise schedule. We experimentally evaluated inference‐step counts (see Supporting Information) and observed that doubling from 200 to 400 steps raises PSNR by only 0.11 dB, yet almost doubles inference time and compute load. In contrast, halving to 100 steps reduces PSNR by roughly 1 dB, a substantial quality loss. Given these trade‐offs between marginal quality gains and sharply increased cost, we choose 200 steps for our final model to balance reconstruction accuracy and computational efficiency.

### 4.6 The Detailed Structure of SC‐Block

Originating from control theory, SSMs [47] have garnered increasing attention due to their efficacy in long sequence modeling. Unlike self‐attention based transformers, SSMs capture long‐range token interactions through linear recurrent processes, entailing O(N) complexity theoretically. Mamba [45, 48] improves the expressiveness of SSMs by introducing a selective mechanism, with its structural parameters adaptively learned from inputs (see supplementary documents for a detailed introduction of State Space Models). In this work, we built our SC‐block based on the Mamba module.

Figure 6b shows the detailed structure of the SC‐block. Assuming that the inputs of SC‐block are feature maps $X_{\mathrm{in}}\in R^{\frac{H}{2^{i}}*\frac{W}{2^{i}}*2^{i}C}$ at different scales. To be specific, for an input feature map $X_{\mathrm{in}}$, it is first passed through a 1×1 convolution and split evenly into two feature maps $X_{1}$ and $X_{2}$,

(18) $$X_{1},X_{2}=\mathrm{Split}\left(\mathrm{Conv}1\times 1\left(X_{\mathrm{in}}\right)\right).$$

Next, $X_{1}\in R^{\frac{H}{2^{i}}*\frac{W}{2^{i}}*2^{i}C}$ is fed into the spatial‐wise SSM module for further processing. Our spatial‐wise SSM module is built on the Select‐Scan Structured State Space for Sequences (S6) block [48] (see supplementary document for the detailed description of the S6 block). The S6 block is the core of Mamba and can achieve global receptive field, dynamic weight, and linear complexity at the same time. While the sequential nature of the scanning operation in S6 aligns well with natural language processing tasks involving temporal data, it poses a significant challenge when applied to vision data, which is inherently nonsequential and encompasses spatial information (e.g., local texture and global structure). To simultaneously perceive local textures and global features in visual data, we propose the multi‐scale SSM layer, which uses a multi‐scale cycle selective‐scan strategy (as shown in Figure 6c) to adapt S6 to vision data without compromising its advantages.

As shown in Figure 6b, the input feature $X_{1}$ will go through two parallel branches. In the first branch, the feature's channel number is expanded to $\lambda 2^{i}C$ by a linear layer, where λ is a pre‐defined channel expansion factor. Subsequently, the feature is processed by a depth‐wise convolution (DWConv) [49] and a multi‐scale SSM layer (as shown in Figure 6c). In the second branch, the features channel is also expanded to $\lambda 2^{i}C$ with a linear layer. The linear layer consists of a 1×1 convolution and a SiLu activation function. After that, features from the two branches are aggregated with the Hadamard product. Finally, the channel number is projected back to $2^{i}C$ to generate output 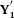 with the same shape as input,

(19)

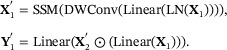




Then, 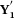 needs to be added with a residual value controlled by the learnable scale factor $\alpha_{1}$ to get the output of spatial‐wise SSM module,

(20)

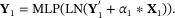




Similarly, $X_{2}\in R^{\frac{H}{2^{i}}*\frac{W}{2^{i}}*2^{i}C}$ is sent to the channel‐wise SSM module. In terms of channel‐wise information modeling, existing Mamba architectures primarily scan images from four spatial directions, without considering dependencies across channels. To address this limitation, we extended the 2D‐SSM [45] to achieve cross‐channel scanning within the Channel‐wise Selective Scan module. As illustrated, the channel‐wise SSM first applies a Global Average Pooling(GAP) operation to the input $X_{2}$ to obtain pooled features. Next, the pooled feature is processed through a SiLU activation function and an SSM layer. Finally, a sigmoid activation function is used to generate the channel attention, which is then multiplied by the original input $X_{2}$ to produce the output of the Channel‐SSM, which is defined as follows,

(21) $$\begin{array}{cc} X_{2}^{\prime } & =\mathrm{Sigmoid}(\mathrm{SSM}\left(\mathrm{SiLU}\left(\mathrm{Conv}\left(\mathrm{GAP}\left(X_{2}\right)\right)\right)\right)),\\ Y_{2}^{\prime } & =X_{2}⊙X_{2}^{\prime }, \end{array}$$

Then, 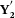 needs to be added with a residual value controlled by the learnable scale factor $\alpha_{2}$ to get the output of the channel‐mamba module,

(22) $$\begin{array}{c} Y_{2}=\mathrm{MLP}\left(\mathrm{LN}\left(Y_{2}^{\prime }+\alpha_{2}*X_{2}\right)\right) \end{array}$$

Finally, $Y_{1}$ and $Y_{2}$ are concatenated as the input of a 1×1 convolution which has a residual connection with the input $X_{in}$. As such, the final output of the SC‐block is given by,

(23) $$\begin{array}{c} X_{out}=\mathrm{Conv}1\times 1\left(\mathrm{Concat}\left(Y_{1},Y_{2}\right)\right)+X_{in}. \end{array}$$

### 4.7 Loss Function

The F‐Principle [50] proves that deep learning networks tend to prefer low frequencies to fit the objective, which will result in the frequency domain gap. Recent studies [51, 52] indicate that the periodic pattern shown in the frequency spectrum may be consistent with the artifacts in the spatial domain. In scattering imaging, the model overfitting at low frequencies brings smooth textures and blurry structures. So exploring adaptive constraints on specific frequencies is essential for the refined reconstruction.

In this work, we use the Discrete Fourier Transform (DFT) to convert the images to the frequency domain and to supervise the frequency distance between truth and predicted images adaptively. We calculate the frequency spectrum for each channel. In a specific channel k, the conversion relationship between spatial coordinates (h,w,k) and frequency domain coordinates (u,v) is expressed as,

(24) $$\begin{array}{cc} F^{k}\left(u,v\right) & =\sum_{h=0}^{H-1}\sum_{w=0}^{W-1}I\left(h,w,k\right)e^{-j2\pi (\frac{uh}{H}+\frac{vw}{W})}, \end{array}$$

where F is the frequency spectrum of all channels corresponding to the image I. Then we can calculate the dynamic weights based on the frequency distance to make the model concentrate on high‐frequency details that are difficult to reconstruct. Specifically, in each channel k, the frequency distance between ground truth and predicted image is equivalent to the power distance between their spectrum $F_{gt}^{k}$ and $F_{pred}^{k}$, which is defined as,

(25) $$\begin{array}{c} d^{k}\left(u,v\right)={\left\|F_{gt}^{k}\left(u,v\right)-F_{pred}^{k}\left(u,v\right)\right\|}^{2}. \end{array}$$

Next, we define a dynamic weight factor θ(u,v) linearly related to the distance d(u,v) to make the model pay more attention to the frequencies that are hard to synthesize. Then the distance between the ground truth and the predicted image in a single channel k is formulated as,

(26) $$\begin{array}{c} d\left(F_{gt}^{k},F_{pred}^{k}\right)=\frac{1}{HW}\sum_{u=0}^{H-1}\sum_{v=0}^{W-1}\theta^{k}\left(u,v\right)d^{k}\left(u,v\right), \end{array}$$

where $\theta^{k}\left(u,v\right)$ changes linearly with the absolute value of the $k_{th}$ channel frequency distance $\sqrt{\left(∥d^{k}\left(u,v\right)∥\right)}$. We traverse k={0,1,2…C−1} and sum each distance to calculate the frequency domain loss $L_{FWL}$ as,

(27) $$\begin{array}{c} L_{FWL}\left(F_{gt},F_{pred}\right)=\sum_{k=0}^{C-1}d\left(F_{gt}^{k},F_{pred}^{k}\right). \end{array}$$

In addition, we also used the spatial‐wise $L_{1}$ loss, and combined it with the $L_{FWL}$ to form a dual domain loss,

(28) $$\begin{array}{c} L_{total}=L_{1}\left(I_{gt},I_{pred}\right)+\lambda L_{FWL}\left(F_{gt},F_{pred}\right), \end{array}$$

where λ is a weight factor, which makes the values of $L_{1}$ and $L_{FWL}$ be of the same order of magnitude.

## Conflicts of Interest

The authors declare no conflicts of interest.

## Supporting information


**Supporting File**: advs75390‐sup‐0001‐SuppMat.pdf.

## Data Availability

All data and code for this work are available at https://github.com/LintaoPeng/ultra‐wide‐field‐Noninvasive‐imaging.
