# Supplementary material for: Ultra‐Wide‐Field Noninvasive Imaging Through Scattering Media Via Physics‐Guided Deep Learning
Source: Adv Sci (Weinh). 2026 May 7;13(40):e75390. doi: 10.1002/advs.75390 (PMC13335759; doi:10.1002/advs.75390)
Supplement: Supplementary file 1 — Supporting File: advs75390‐sup‐0001‐SuppMat.pdf. [file ADVS-13-e75390-s001.pdf]

Supplementary Materials for Ultra-wide-field Noninvasive Imaging through Scattering Media via Physics-guided Deep Learning

Lintao Peng Mingwei He Jeff Zhu Sujit K. Sahoo Liheng Bian* Cuong Dang**

Lintao Peng, Mingwei He, Jeff Zhu, Cuong Dang
School of Electrical and Electronic Engineering, Nanyang Technological University,Singapore, 639798, Singapore
Email Address:hcdang@ntu.edu.sg
Lintao Peng, Liheng Bian
State Key Laboratory of Environment Characteristics and Effects for Near-space & State Key Laboratory of CNS/ATM, Beijing Institute of Technology, Beijing, 100081, China
Email Address:bian@bit.edu.cn
Sujit K. Sahoo
School of Electrical Sciences, Indian Institute of Technology Goa, Goa, 403401, India
Email Address:sujit@iitgoa.ac.in

This supplementary material accompanies the paper titled "Ultra-wide-field Noninvasive Imaging through Scattering Media via Physics-guided Deep Learning." In this supplementary material, we present additional visual results for scattering imaging experiments, demonstrate imaging performance exceeding one hundred times the optical memory effect (OME) range, and perform experiments to assess the necessity of pre-training. We further investigate the relationship between the number of diffusion steps in our UNI-Net and imaging performance, clarifying the rationale for selecting 200 steps. In addition, we provide the training details of the proposed UNI-Net, the specifications of the optical setup, and a detailed description of the state space model.

1 Supplementary Material for Scattering Imaging Performance Comparison

To further demonstrate the capability of our approach to reconstruct complex scenes and evaluate its performance on datasets with multiple classes and high structural complexity, we present additional comparative scattering imaging results under complex environments using the Fashion-MNIST [1] and CIFAR [2] benchmarks. Fashion-MNIST consists of diverse clothing items, while CIFAR includes natural images characterized by richer textures and more complex object structures. For all speckle acquisitions, the displayed scenes were fixed at a resolution of 32×32 pixels. To ensure fairness in comparison, all learning-based methods were pre-trained on the same dataset. For each dataset, the models were pre-trained on 10,000 simulated speckle–image pairs and subsequently fine-tuned on 2,000 experimentally acquired pairs.
In Fig. S1, we present supplementary visual scattering imaging results. The comparison includes IDiffNet [3], PDSNet [4], NSDN [5], and the proposed method. As shown in Fig. S1, existing methods encounter difficulties in reconstructing complex scenes. They recover only coarse global structures, fail to reconstruct detailed textures and sharp edges, and frequently introduce artifacts. In contrast, our UNI-Net accurately restores scene structures and faithfully reconstructs texture-rich regions and sharp edges. This superior performance is attributed to three key innovations. First, the full speckle pattern is partitioned into multichannel speckle patches, which are used as guidance in the diffusion model's iterative denoising process, thereby maximizing the utilization of speckle information. Second, the proposed SC-block offers a global spatial receptive field and models long-range inter-channel dependencies with linear computational complexity, thereby enabling the recovery of fine-grained structures in complex images. Finally, the FWL loss function strengthens the network's focus on challenging texture-rich and edge regions, further enhancing image quality.

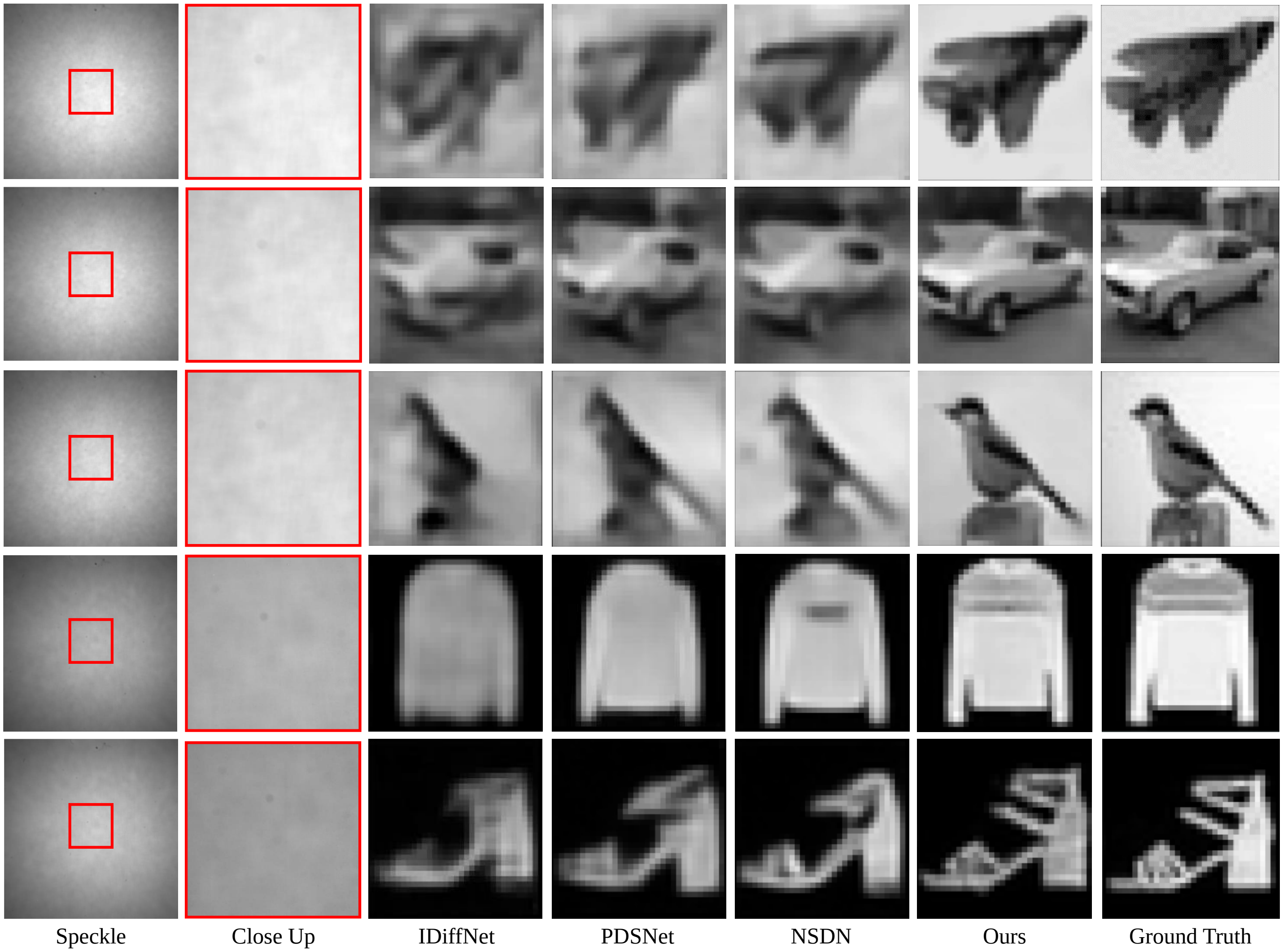

Figure S 1: Supplementary visualization of scattering imaging performance in complex scenes is provided. We conducted comparative experiments on complex scenes with the CIFAR-10 and Fashion-MNIST datasets. The reconstruction results of several baseline methods (IDiffNet, PDSNet, and NSDN), along with those of our method, are presented. All methods were initially pretrained on the same simulated dataset and subsequently fine-tuned on a real dataset to ensure a fair comparison.

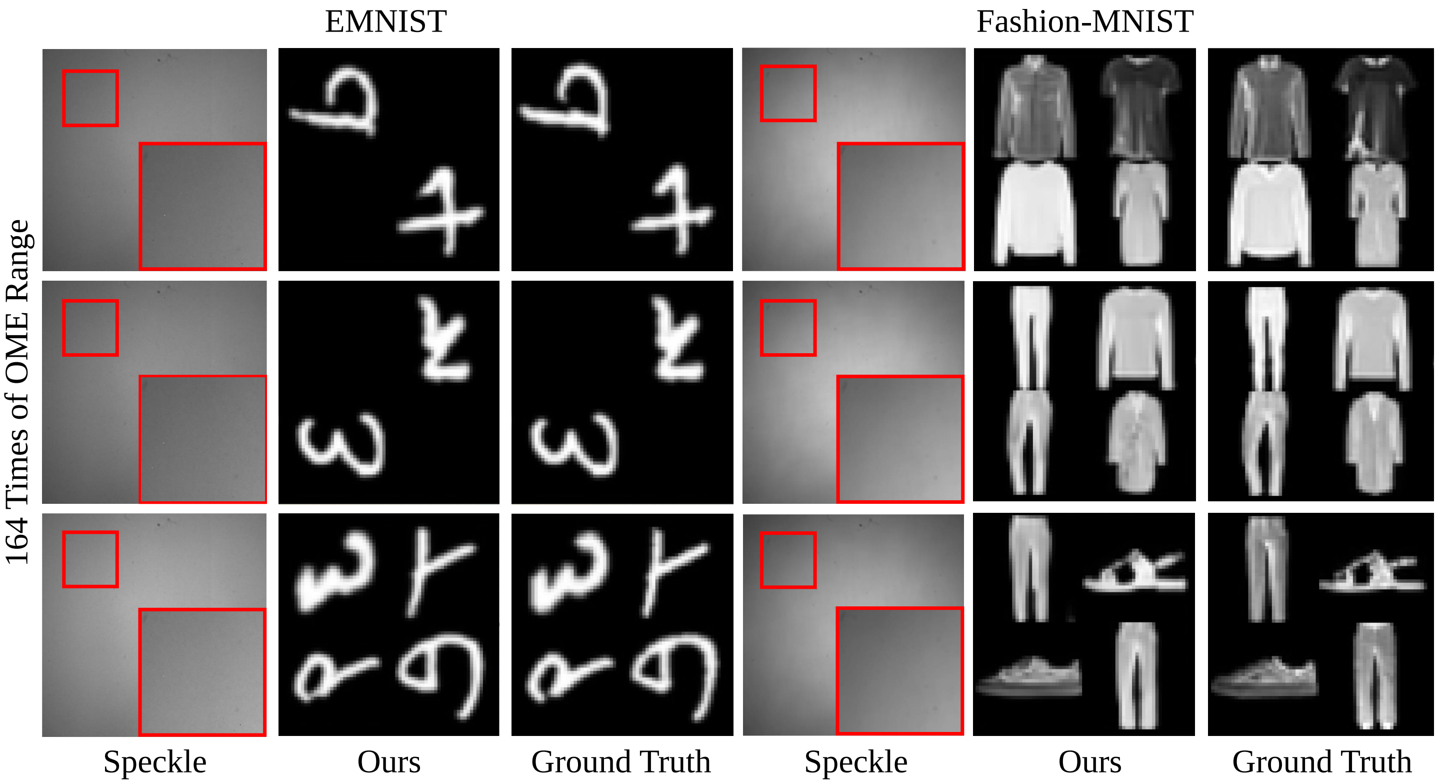

Figure S 2: Quantitative comparison of scattering imaging performance beyond the optical memory effect (OME) range is presented. We conducted scattering imaging experiments at 164 times beyond the OME range on the EMNIST and Fashion-MNIST datasets, respectively. The image resolution at 164 times beyond the OME range was 128 $\times$ 128 pixels.

2 Supplementary Material for Imaging Beyond Hundred Times of OME Range

To further demonstrate the imaging performance of the proposed UNI-Net at 164$\times$ the OME range, we present additional visual scattering imaging results under this extreme condition. As shown in Fig. S2, under the extreme 164$\times$ OME range, the proposed method successfully recovers the global structure of the target scene and faithfully reconstructs its local details. This superior performance is attributed to the two-stage approach of pre-training on simulated data followed by fine-tuning on real measurements, which substantially enhances model robustness under ultra-wide-field conditions. This also demonstrates that incorporating speckle intensity as physical supervision in each iteration of the diffusion model's denoising process effectively improves reconstruction fidelity. Moreover, partitioning the acquired speckle pattern into multi-channel blocks substantially improves information utilization. Overall, the integration of simulation-based pre-training with multi-channel speckle–guided iterative denoising is central to the superior performance of the proposed method.

3 Supplementary Materials for Experiments on the Necessity of Pre-training

To evaluate the effectiveness of the proposed physical scattering imaging model and the pre-training strategy, comparative experiments were conducted under two conditions: with and without pre-training. In the pre-training condition, the proposed UNI-Net was first pre-trained on a simulated dataset and subsequently fine-tuned using varying amounts of real speckle–ground truth image pairs. In contrast, in the absence of pre-training, the model was trained from scratch using the same amount of real-world data. The experimental results are summarized in Tab. S1.
As shown in Tab. S1, pre-training on simulated data substantially enhances the model's reconstruction performance while reducing its reliance on real data. For instance, the pre-trained model achieved a PSNR

Table S 1: Comparison of reconstruction performance (PSNR (dB)) with and without pre-training under varying amounts of real data. The "With Pre." setting refers to models pre-trained on 10,000 simulated speckle–ground truth pairs and fine-tuned on varying amounts of real data, while "W/O Pre." refers to models trained solely on real data without pre-training. Results demonstrate that pre-training significantly improves reconstruction quality and reduces the need for real experimental data by an order of magnitude.

Method	Index	100	200	300	400	500	700	900	1100	1300	1500	1800	2400
With Pre. on EMNIST	PSNR	20.76	24.94	28.19	31.67	33.31	34.43	35.67	36.06	36.09	36.08	36.13	36.25
	SSIM	0.596	0.686	0.737	0.818	0.865	0.896	0.931	0.941	0.942	0.941	0.944	0.949
W/O Pre. on EMNIST	PSNR	13.04	14.46	15.55	16.13	16.69	17.17	18.03	19.15	19.93	20.64	21.84	24.36
	SSIM	0.387	0.416	0.432	0.443	0.464	0.482	0.504	0.531	0.542	0.563	0.605	0.679
With Pre. on CIFAR	PSNR	16.79	19.43	21.17	22.27	23.08	24.96	26.73	27.67	28.69	29.28	29.31	29.41
	SSIM	0.523	0.605	0.639	0.673	0.698	0.756	0.812	0.841	0.873	0.891	0.892	0.895
W/O Pre. on CIFAR	PSNR	10.35	11.73	12.67	13.43	14.17	15.32	16.27	17.03	17.74	18.29	19.07	20.09
	SSIM	0.322	0.365	0.394	0.418	0.441	0.474	0.506	0.529	0.552	0.569	0.593	0.625

of 24.94 dB using only 200 real image pairs, whereas the model trained from scratch required 2,400 pairs to reach comparable performance, thereby reducing the demand for real data by an order of magnitude.

4 Diffusion Steps and Efficiency Trade-off

Although diffusion-based models can achieve high-quality reconstructions through iterative refinement of noisy signals, the number of denoising steps T is a critical factor that influences both performance and efficiency. A larger T generally yields improved image quality but results in longer inference time and greater memory consumption. To address this issue, we adopt the approach in Ref. [6] as the inference sampler, which enables flexible and efficient control over the number of denoising steps without requiring retraining. This section investigates the impact of different inference step counts on reconstruction quality and inference cost, with the aim of identifying an optimal balance suitable for practical deployment.
We evaluate our proposed method on the EMNIST dataset and Fashion MNIST dataset with the number of inference steps $T \in (29, 50, 100, 200, 400, 600, 800)$. all methods were first pre-trained on the same simulated dataset, then fine-tuned on the same real dataset, and finally evaluated on the same test set. We report the average PSNR and SSIM over multiple test samples, along with the average inference time(S) and computational cost (GFLOPs) per image using an NVIDIA RTX 4090 GPU. The model weights and architecture remain fixed across all configurations to ensure consistency.
Tab. S2 reports the quantitative performance and computational cost at different diffusion step counts. As expected, increasing inference steps T leads to gradual improvement in PSNR and SSIM, but the gain saturates beyond T $=$ 200. Specifically, increasing the number of inference steps from 200 to 400 only yields a marginal PSNR gain of 0.11 dB, while nearly doubling the inference time and memory consumption. Conversely, reducing the step count from 200 to 100 significantly degrades reconstruction quality by $\sim$ 1.0 dB in PSNR. Our analysis reveals that T $=$ 200 provides a sweet spot where the model achieves high fidelity with moderate inference cost.

5 Implementation and Training Details

The methods included in the comparative experiments are SVR [7], IDiffNet [3], PDSNet [4], DeepSCI [8], NSDN [5], and the proposed method. For DeepSCI, the authors' publicly released code was employed. For SVR, IDiffNet, PDSNet, and NSDN, their machine learning or deep learning models were reimple-

Table S 2: Quantitative evaluation of reconstruction quality (PSNR in dB / SSIM), average inference time per image in seconds, and computational cost (GFLOPs) across different inference step counts T on EMNIST and Fashion MNIST datasets. Quality gains plateau beyond $T = 200$, while inference and imaging times increase markedly.

Steps		20	50	100	200	400	600	800
EMNIST	PSNR	31.48	33.12	35.36	36.25	36.36	36.38	36.41
	SSIM	0.851	0.896	0.931	0.949	0.952	0.952	0.952
Fashion	PSNR	28.17	30.16	32.64	33.79	33.91	33.89	33.93
	SSIM	0.801	0.834	0.907	0.922	0.926	0.925	0.926
Time(s)		4.26	5.72	7.07	12.93	21.48	29.26	38.76
Imaging Time(s)		0.032	0.041	0.054	0.087	0.158	0.217	0.296

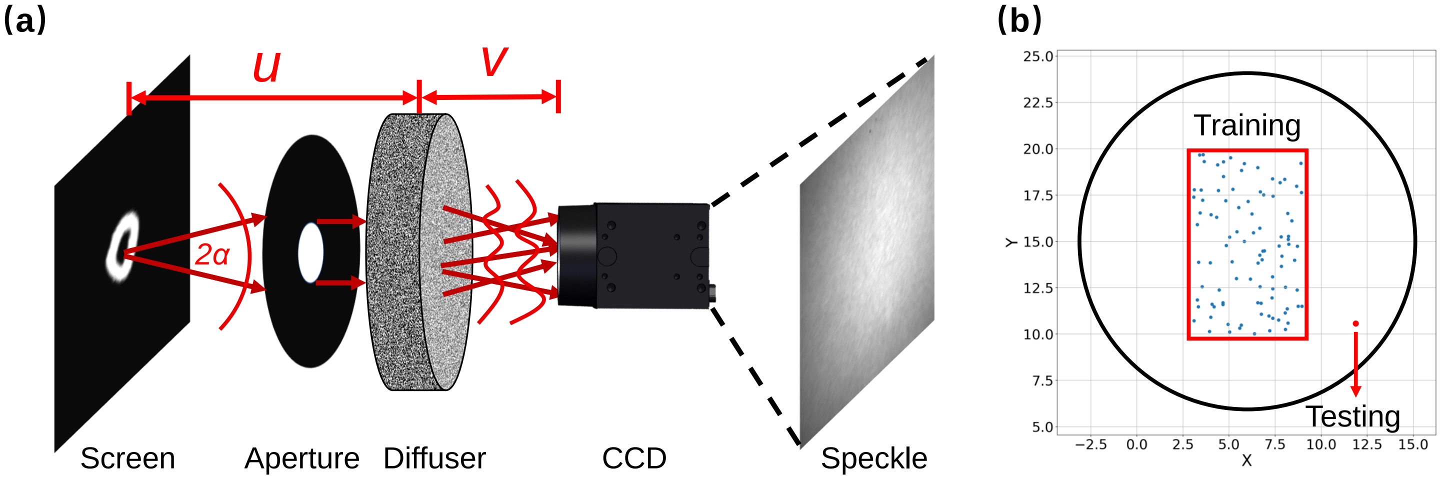

Figure S 3: **a.** Proof-of-concept optical setup. **b.** Regions on the diffuser used for speckle-pattern acquisition. Blue dots within the red rectangle indicate diffuser positions used for training-set collection, while red dots indicate positions used for test-set collection.

mented in Python using the PyTorch framework, following the network architectures described in the original papers. Before conducting the comparative experiments, each reproduced method was trained and tested under the experimental settings specified in its respective paper to confirm that the reported imaging performance was achieved. All methods were trained and evaluated using the same hardware (Intel Core i9-14900K, NVIDIA RTX4090, 64 GB RAM) and software environment (Windows 11, Python 3.8.10, PyTorch 1.12.0). The original hyperparameter settings for each method were adhered to, and the networks were trained until their loss functions converged.

The proposed UNI-Net was implemented on Ubuntu 20 using the PyTorch framework and trained with the Adam optimization algorithm on an NVIDIA RTX 4090. The proposed method and the comparative methods were trained on the EMNIST, Fashion-MNIST, and CIFAR datasets. The training sets were augmented by rotation and flipping of images. All images were normalized to the [0,1] range before being input into the networks. During training, the batch size was set to 16, and the proposed UNI-Net was trained for a total of 1,500 epochs. The Adam optimizer was used for optimization. The initial learning rate was set to 0.0001 and decayed by a factor of 0.8 every 300 iterations. For efficient restoration, the time step T was set to 200 during both training and testing. The default values for β_1 and β_2 were set to 0.5 and 0.999, respectively. The weight decay was set to 0.00005.

6 Details about Our Optical Setup

In this section, we provide a detailed explanation of how the magnification factor is calculated and how the numerical aperture (NA) is measured in our proof-of-concept setup. We also describe in detail how we avoid the overlap between the PSFs of the training and testing sets during speckle pattern acquisition.

As illustrated in Fig. S3a, the target scene is rendered on an OLED screen, and the light first passes through a pinhole aperture, spatially confining the beam and suppressing stray illumination. Subsequently, the beam traverses a diffuser mounted on a motorized stage that undergoes randomized translations, generating a unique point spread function (PSF) for each acquisition and thereby enriching the dataset. Finally, the scattered light is captured by a CCD detector, and the recorded speckle patterns are input to our model for high-quality image reconstruction.

In our optical setup, the distance from the CCD to the scattering plate is denoted by v, and the distance from the scattering plate to the OLED display is u; hence the system magnification M is given by $M = v/u$. To measure the NA, we denote by 2α the angular extent subtended by the system's aperture at the object plane, so that $NA = sin(\alpha)$. Accordingly, the system cutoff frequency is,

$$F_{\text{cut-off}} = \frac{2\pi \sin\alpha}{\lambda} = \frac{2\pi \text{ NA}}{\lambda}, \tag{1}$$

and the optical resolution is defined as,

$$\text{Resolution} = \frac{\pi}{F_{\max}} = \frac{\pi}{F_{\text{cut-off}}} = \frac{\lambda}{2\sin\alpha} = \frac{\lambda}{2 \text{ NA}}. \tag{2}$$

To ensure the PSFs between training and test data are non-overlapping, speckle patterns for network training were sampled within a rectangular region on the diffuser during speckle-pattern acquisition, while patterns for performance evaluation were acquired outside this rectangle, as illustrated in Fig. S3b. Note that this schematic illustration does not correspond to the exact diffuser positions. In actual data acquisition, it suffices to ensure that the PSF sampling regions for the training and testing sets do not overlap.

7 Detail Introduction of State Space Models

Originating from control theory, State Space Models (SSMs)[9] have garnered increasing attention due to their efficacy in long-term language modeling. Unlike self-attention based transformers, most SSMs capture long-range token interactions through linear recurrent processes, entailing $\mathcal{O}(N)$ complexity theoretically. Mamba [10, 11] improves the expressiveness of SSMs by introducing a selective mechanism, with its structural parameters adaptively learned from inputs. Motivated by its potential for modeling high-resolution images, many researchers try to apply Mamba to vision tasks. For instance, VMamba [12] introduces a cross-scan module to enable 1D selective scanning in 2D image space. LocalMamba [13] utilizes local windows to enhance local modeling capability. EfficientVMamba [14] designs an atrous-based selective scan approach to enhance efficiency. In this work, we propose an multi-scale cycle selective scan module based on the existing Mamba, which facilitates the gathering of local details while obtaining the global receptive field with linear complexity.

In a typical Mamba model, SSMs map input sequence $x(t) \in \mathbb{R}$ to output sequence $y(t) \in \mathbb{R}$ through hidden state $h(t) \in \mathbb{R}^N$. They set linear mappings $\mathbf{A} \in \mathbb{R}^{N\times N}$, $\mathbf{B} \in \mathbb{R}^{N\times 1}$ and $\mathbf{C} \in \mathbb{R}^{1\times N}$ as transition parameters to update the hidden state over time and obtain the output. The mapping process is as follows,

$$\begin{aligned} h'(t) &= \mathbf{A}h(t) + \mathbf{B}x(t), \\ y(t) &= \mathbf{C}h(t). \end{aligned} \tag{3}$$

To enable network training, recent SSM such as Mamba [10] discretize the above process through an extra timescale parameter Δ. Then, continuous parameters $\mathbf{A}$ and $\mathbf{B}$ are transformed into the discretized

ones $\overline{\mathbf{A}}$ and $\overline{\mathbf{B}}$ via the zero-order hold (ZOH) method,

$$\begin{aligned}\overline{\mathbf{A}} &= \exp(\Delta\mathbf{A}),\\ \overline{\mathbf{B}} &= (\Delta\mathbf{A})^{-1}(\exp(\Delta\mathbf{A}) - \mathbf{I})\Delta\mathbf{B},\end{aligned} \tag{4}$$

where $\mathbf{I}$ denotes the identity matrix and $(\cdot)^{-1}$ is the matrix inversion operation. Therefore, we now derive the discretized version of E.q. 1,

$$\begin{aligned}h_t &= \overline{\mathbf{A}}h_{t-1} + \overline{\mathbf{B}}x_t,\\ y_t &= \mathbf{C}h_t.\end{aligned} \tag{5}$$

The above recurrent form can be deviated into a convolutional form with global kernel $\mathbf{K}$ to enable parallel training,

$$\begin{aligned}\overline{\mathbf{K}} &= (\mathbf{C}\overline{\mathbf{B}}, \mathbf{C}\overline{\mathbf{AB}}, ..., \mathbf{C}\overline{\mathbf{A}}^{L-1}\mathbf{B}),\\ \mathbf{y} &= \mathbf{x} * \overline{\mathbf{K}},\end{aligned} \tag{6}$$

where L denotes the sequence length and $*$ represents the convolution operator. Moreover, Mamba introduces a selective mechanism on $\overline{\mathbf{B}}$ and $\mathbf{C}$ to enable context-aware information filtering and a parallel scan algorithm [10] to speed up the process in E.q. 5 for faster training. The above is the detailed structure and calculation process of an Select-Scan Structured State Space for Sequences (S6) block [10]. Our MCSS module is built on the S6 block [10]. The S6 block is the core of Mamba and can achieve global receptive field, dynamic weight, and linear complexity at the same time. While the sequential nature of the scanning operation in S6 aligns well with NLP tasks involving temporal data, it poses a significant challenge when applied to vision data, which is inherently non-sequential and encompasses spatial information (e.g., local texture and global structure). In order to simultaneously perceive local textures and global features in visual data, we propose the multi-scale cycle selective-scan strategy (as shown in Fig. 6(c) in the manuscript) to adapt S6 to vision data without compromising its advantages.

References

[1] H. Xiao, K. Rasul, R. Vollgraf, Fashion-mnist: a novel image dataset for benchmarking machine learning algorithms. *arXiv preprint arXiv:1708.07747* (2017).

[2] A. Krizhevsky, G. Hinton, *et al.*, Learning multiple layers of features from tiny images.(2009) (2009).

[3] S. Li, M. Deng, J. Lee, A. Sinha, G. Barbastathis, Imaging through glass diffusers using densely connected convolutional networks. *Optica* **5**, 803–813 (2018).

[4] E. Guo, S. Zhu, Y. Sun, L. Bai, C. Zuo, J. Han, Learning-based method to reconstruct complex targets through scattering medium beyond the memory effect. *Opt. Express* **28**, 2433–2446 (2020).

[5] S. Yan, Y. Sun, F. Ni, Z. Liu, H. Liu, X. Chen, Image reconstruction through a nonlinear scattering medium via deep learning. *Photonics Res.* **12**, 2047–2055 (2024).

[6] Y. Song, J. Sohl-Dickstein, D. P. Kingma, A. Kumar, S. Ermon, B. Poole, *ICLR*.

[7] R. Horisaki, R. Takagi, J. Tanida, Learning-based imaging through scattering media. *Opt. Express* **24**, 13738–13743 (2016).

[8] Z. Tang, F. Wang, Z. Fu, S. Zheng, Y. Jin, G. Situ, Deepsci: scalable speckle correlation imaging using physics-enhanced deep learning. *Optics Letters* **48**, 2285–2288 (2023).

[9] A. Gu, K. Goel, C. Ré, Efficiently modeling long sequences with structured state spaces. *arXiv preprint arXiv:2111.00396* (2021).

[10] A. Gu, T. Dao, Mamba: Linear-time sequence modeling with selective state spaces. *arXiv preprint arXiv:2312.00752* (2023).

[11] R. Xu, S. Yang, Y. Wang, B. Du, H. Chen, A survey on vision mamba: Models, applications and challenges. *arXiv preprint arXiv:2404.18861* (2024).

[12] Y. Liu, Y. Tian, Y. Zhao, H. Yu, L. Xie, Y. Wang, Q. Ye, Y. Liu, Vmamba: Visual state space model. *arXiv preprint arXiv:2401.10166* (2024).

[13] T. Huang, X. Pei, S. You, F. Wang, C. Qian, C. Xu, Localmamba: Visual state space model with windowed selective scan. *arXiv preprint arXiv:2403.09338* (2024).

[14] X. Pei, T. Huang, C. Xu, Efficientvmamba: Atrous selective scan for light weight visual mamba. *arXiv preprint arXiv:2403.09977* (2024).
